# On the reproducibility of meta-analyses: six practical recommendations

**DOI:** 10.1186/s40359-016-0126-3

**Published:** 2016-05-31

**Authors:** Daniël Lakens, Joe Hilgard, Janneke Staaks

**Affiliations:** Human Technology Interaction Group, Eindhoven University of Technology, IPO 1.33, PO Box 513, 5600MB Eindhoven, The Netherlands; University of Missouri, Missouri, USA; University of Amsterdam, Amsterdam, The Netherlands

**Keywords:** Meta-analysis, Reproducibility, Open science, Reporting guidelines

## Abstract

**Background:**

Meta-analyses play an important role in cumulative science by combining information across multiple studies and attempting to provide effect size estimates corrected for publication bias. Research on the reproducibility of meta-analyses reveals that errors are common, and the percentage of effect size calculations that cannot be reproduced is much higher than is desirable. Furthermore, the flexibility in inclusion criteria when performing a meta-analysis, combined with the many conflicting conclusions drawn by meta-analyses of the same set of studies performed by different researchers, has led some people to doubt whether meta-analyses can provide objective conclusions.

**Discussion:**

The present article highlights the need to improve the reproducibility of meta-analyses to facilitate the identification of errors, allow researchers to examine the impact of subjective choices such as inclusion criteria, and update the meta-analysis after several years. Reproducibility can be improved by applying standardized reporting guidelines and sharing all meta-analytic data underlying the meta-analysis, including quotes from articles to specify how effect sizes were calculated. Pre-registration of the research protocol (which can be peer-reviewed using novel ‘registered report’ formats) can be used to distinguish a-priori analysis plans from data-driven choices, and reduce the amount of criticism after the results are known.

**Summary:**

The recommendations put forward in this article aim to improve the reproducibility of meta-analyses. In addition, they have the benefit of “future-proofing” meta-analyses by allowing the shared data to be re-analyzed as new theoretical viewpoints emerge or as novel statistical techniques are developed. Adoption of these practices will lead to increased credibility of meta-analytic conclusions, and facilitate cumulative scientific knowledge.

## Basckground

### On the reproducibility of meta-analyses: six practical recommendations

Any single study is just a data-point in a future meta-analysis. Meta-analyses play an important role in cumulative science by combining information across multiple studies. Especially when sample sizes are small, effect size estimates can vary substantially from one study to the next. Statistical inferences are even more complicated due to publication bias. Because statistically significant studies are more likely to be published, it is a challenge to draw quantifiable conclusions about the presence or absence of effects based on the published literature. Meta-analytic statistical tools can provide a partial solution to these problems by drawing conclusions over multiple independent studies and calculating effect size estimates that attempt to take publication bias into account. Although meta-analyses do not provide definitive conclusions, they are typically interpreted as state-of-the-art empirical knowledge about a specific effect or research area. Large-scale meta-analyses often accumulate a massive number of citations and influence future research and theory development. It is therefore essential that published meta-analyses are of the highest possible quality.

To guarantee this quality, both pre- and post-publication peer review are important tools. Peers can check the quality of meta-analyses only when the meta-analytic effect size calculations are reproducible. Unfortunately, this is rarely the case. Gøtzsche et al. [30] examined whether standardized mean differences in meta-analyses were correctly calculated and reproducible for 27 meta-analyses. In 10 (37 %) of these studies, results could not be reproduced within a predefined difference of the effect size estimate of 0.1. Among these ten meta-analyses, one that had yielded a significant meta-analytic effect was no longer significant, three without significant results yielded significant results when reproduced, and one meta-analysis was retracted. While quality control is important, there are other benefits to reproducible meta-analyses. When meta-analyses are well-documented and transparent, it is easier to re-analyze published meta-analyses using different inclusion criteria. Such re-analyses can yield important insights that differ from the conclusions in the initial meta-analysis. For example, Derry et al. [20] found that even though many systematic reviews had concluded acupuncture was beneficial, their meta-analysis using stricter inclusion criteria (such as randomized, double blind trials) revealed strong evidence that acupuncture had no benefit.

Beyond re-analyses, making meta-analytic data publically available facilitates continuously cumulating meta-analyses that update effect size estimates as new data are collected (e.g., [7]) or as new theoretical viewpoints or statistical techniques are developed. Meta-analyses need to be updated regularly to prevent outdated scientific conclusions from influencing public policy or real-life applications. Cochrane reviews are required to be updated every 2 years, or else the lack of an update needs to be justified [17]. Similarly, authors of Campbell reviews are obliged to plan for an update within 5 years after publication [12]. If the data underlying meta-analyses were openly accessible and reproducible, such updates may become more common in psychology than they are now, which would facilitate cumulative knowledge. If meta-analytic efforts are to stand the test of time, it is essential that the extracted meta-analytic data can be easily updated and reanalyzed by researchers in the future.

The open accessibility of meta-analytic data may also make these analyses more objective and convincing. A lack of openness about the data and choices for inclusion criteria underlying meta-analyses has been raised as one of the problems in resolving debates following the publication of meta-analyses (e.g., [10]). As Ferguson [23] argues, “meta-analyses have failed in replacing narrative reviews as more objective”. Researchers on different sides of a scientific argument often reach different conclusions in their meta-analyses of the same literature (see [28], for detailed discussion of different meta-analyses on depression screening with opposite recommendations). We believe this situation can be improved substantially by implementing six straightforward recommendations that increase the openness and reproducibility of meta-analyses. We have summarized these recommendations in Table 1, and will expand upon each in the remainder of this article.

**Table 1:** Six practical recommendations to improve the reproducibility of meta-analyses

1. Facilitate cumulative science by future-proofing meta-Analyses: Disclose all meta-analytic data (effect sizes, sample sizes for each condition, test statistics and degrees of freedom, means, standard deviations, and correlations between dependent observations) for each data point. Quote relevant text from studies that describe the meta-analytic data to prevent confusion, such as when one effect size is selected from a large number of tests reported in a study. When analyzing subgroups, include quotes from the original study that underlie this classification, and specify any subjective decisions.	
2. Facilitate quality control: Specify which effect size calculations are used and which assumptions are made for missing data (e.g., assuming equal sample sizes in each condition, imputed values for unreported effect sizes), if necessary for each effect size extracted from the literature. Specify who extracted and coded the data, knowing it is preferable that two researchers independently extract effect sizes from the literature.	
3. Adhere to reporting guidelines: A minimal requirement when reporting meta-analyses is to adhere to one of the reporting standards (e.g., PRISMA). The reporting guidelines ask authors of meta-analyses to report essential information that should be made available either in the main text of the article, or by providing a completed checklist as supplementary material during review and after publication.	
4. Pre-register: Whenever possible, pre-register the meta-analysis research protocol to distinguish between confirmatory and exploratory analyses.	
5. Facilitate reproducibility: Allow others to re-analyze the data to examine how sensitive the results are to subjective choices such as inclusion criteria. Always include a link to data files that can be directly analyzed with statistical software, either by providing completely reproducible scripts containing both the data and the reported analyses in free software (e.g., R), or at the very minimum a spreadsheet that contains all meta-analytic data that can easily analyzed in any statistical program.	
6. Recruit expertise: Consider consulting a librarian before you start the literature search, and a statistician before coding the effect sizes, for advice on how make the literature search and effect size calculations reproducible.	

## Facilitate cumulative science by future-proofing meta-analyses

The data underlying meta-analyses consists of the empirical findings from individual studies. Meta-analyses are now commonly based on effect sizes, although novel techniques (such as *p*-curve analysis, [55]) require the test statistics of the effect of interest, not the effect size. Future developments (e.g., a more widespread use of Bayesian meta-analytic techniques) might similarly require test statistics (e.g., *t*-values, *F*-values, and degrees of freedom) instead of effect sizes when performing meta-analyses. Therefore, meta-analytic data should be defined more broadly than just effect sizes and their confidence intervals, and includes sample sizes per condition, means, standard deviations, test statistics, the type of design (within or between subjects), and for within designs the correlations between dependent observations. All this data, together with the calculated effect size and its variance, should be considered the minimum meta-analytic data that is shared with a publication.

We believe the main effort a meta-analyst should be applauded for is collecting and organizing meta-analytic data extracted from the empirical literature. When meta-analyses are performed on a set of small exploratory studies, the meta-analysis chiefly serves to highlight effects that deserve to be examined in larger pre-registered studies. While one might expect larger meta-analyses to provide definitive conclusions, there is often heterogeneity in the effect sizes included in large meta-analyses. This heterogeneity can be examined by stratifying analyses based on characteristics of studies included in the meta-analysis. These sub-group analyses often inspire new theories which require additional data, to be evaluated in future meta-analyses. A meta-analysis that provides support for an effect will often intensify research on a specific topic, both by researchers who aim to use the effect to examine novel hypotheses as by more skeptical researchers who want to examine possible confounds or alternative theoretical explanations.

For these reasons, a meta-analysis is not definitive, but rather represents a temporary assessment of a research area that may be further explored in future meta-analyses, which are greatly facilitated by the availability of data from earlier meta-analyses. Our recommendation to facilitate cumulative science mirrors recent suggestions to improve the disclosure of data in empirical manuscripts (e.g., [14]) as well as disclosure recommendations in new meta-analytic techniques such as *p*-curve analyses [55]. Whenever meta-analysts have contacted the authors of original manuscripts with requests for additional data or analyses and use these in their meta-analysis, it is recommended to ask the original authors for permission to share this meta-analytic data not reported in the original article. Similarly, authors of a meta-analysis should attempt to make (summaries of) unpublished manuscripts or unpublished datasets available (e.g., as supplementary material).

The archival and availability of data allows new statistical techniques to be applied to old meta-analytic data. The past decade new statistical approaches have been developed to more accurately assess and potentially correct for the degree of publication bias in meta-analytic effect size estimates. Each successive technique has offered incremental improvement over previous techniques, which highlight the importance of *future-proofing* meta-analyses.

An early approach to the problem of publication bias was to estimate the *fail-safe N*, the number of null and unpublished studies that would be needed to reduce an effect to non-significance. Fail-safe *N* has since been recognized as a statistical tool with extremely limited usefulness; its assumption that unpublished studies have an average effect size of zero is likely to be violated, and bias in effect size estimation may be caused by selective reporting of analyses within studies rather than the censorship of entire studies (see [34, 48, 50, 55]).

A more recent approach is to apply the *trim-and-fill method*, which attempts to correct for publication bias by inspecting the funnel plot of observed effect sizes and precisions. The trim-and-fill method “trims” small studies at the extremes of the effect size estimate, re-estimates the effect size, and “fills” the funnel plot with inferred studies that might be missing due to publication bias. However, when publication bias is induced by a *p*-value boundary, rather than an effect size boundary, and there is considerable heterogeneity in the effects included in the meta-analysis, the trim-and-fill method might not perform well enough to yield a corrected meta-analytic effect size estimate that is close to the true effect size [45, 59].

More recently, regression-based approaches have been proposed to provide better bias-adjusted meta-analytic effect size estimates [56]. In general, these meta-regression approaches examine the association between the effect size of the studies and their precision, and use this association to estimate a corrected effect size. Researchers have recently started to re-analyze past meta-analyses using these novel statistical techniques and sometimes found that, after controlling for publication bias, there is no longer meta-analytical support for the effect under examination (e.g., see [11]). Future-proofing meta-analyses by making sure their data is available and reproducible will make it easy to re-evaluate meta-analyses when new statistical techniques are developed.

In addition to correcting for publication bias, a second future change we might expect is a switch from the current focus on null-hypothesis significance testing in meta-analyses towards Bayesian inferences [57]. As mentioned earlier, such Bayesian inferences might require researchers to have access to the test statistics (e.g., *t*-values) instead of effect sizes (e.g., see [49]). These statistics are typically not included in meta-analytic reports, nor are they recommended in preliminary suggestions for reporting standards by the APA (e.g., [37]). To future-proof a meta-analysis, authors need to be more thorough than current reporting standards recommend.

## Open meta-analytic data facilitates quality control

A meta-analysis requires the extraction of a large amount of statistics and the careful coding of study characteristics, based on specific inclusion criteria. When meta-analytic data is openly available, readers can check whether the inclusion criteria were properly implemented, the statistics from each study were correctly extracted, and study characteristics were accurately coded. This validation process is important because errors of at least four different types are common in meta-analyses. A first type of error is the *erroneous calculation of effect sizes*. Although large scale attempts to reproduce meta-analyses are limited, researchers who have reproduced published meta-analyses have found errors in 59 % [36], 63 % [30] and even 100 % [26] of the examined meta-analyses. Error rates seem to be unaffected by the experience researchers have with data-extraction [33].

A second type of error is the *inconsistent calculation of effect sizes*. Researchers often have to choose between including an effect size for a simple effect or an interaction. This choice should be consistent across studies, but often is not. For example, assume researcher A publishes a paper revealing slower reaction times on a speeded categorization task after participants have performed a cognitively demanding task, compared to participants who did not have to perform a cognitively demanding task. Researcher B published a similar article using a similar method, but instead of finding an overall effect on the reaction times in the categorization task, only observes a statistically significant effect in the second half of the trials, but not in the first half (leading to an interaction between the manipulation and block). Researcher C publishes a meta-analysis. From the first study, the main effect of the manipulation is included in the meta-analysis, but from the second study, the simple effect from the second block is included instead of the main effect across blocks. The effect size calculation is inconsistent across studies. Either the main effect should be included, or the differences in the second block are included. Regardless of the final choice, the choice should be consistent across studies to examine the meta-analytic support for either hypothesis.

A third type of error is the *incorrect inclusion of effect sizes*. Inclusion criteria are often subjective, but at other times, inclusion criteria are objectively wrong. For example, Gorin et al. [29] performed a meta-analysis of psychosocial interventions to reduce pain in patients with cancer. The authors included three effect sizes in their meta-analyses from [61–63]. A careful look at these three publications reveals they are based on the same data collected from the same 313 participants. Because the three effect size estimates are not independent, only one of the three should be included in the meta-analysis. If researchers would have provided all the meta-analytic data with the publication, the fact that all three publications were based on the data of 313 participants might have been noticed.

A fourth error that can be made is calculating the *incorrect meta-analytic effect size*. Researchers have to choose between a fixed effect model or a random effects model when performing a meta-analysis. Fixed effect models assume a single true effect size underlies all the studies included in the meta-analysis Fixed effect models are therefore only appropriate when all studies in the meta-analysis are practically identical (e.g., use the same manipulation) and when researchers do not want to generalize to different populations [5]. By contrast, random effects models allow the true effect size to vary from study to study (e.g., due to differences in the manipulations between studies). Random effects models therefore are appropriate when a wide range of different studies is examined and there is substantial variance between studies in the effect sizes. Since the assumption that all effect sizes are identical is implausible in most meta-analyses random effects meta-analyses are generally recommended [5]. Using the wrong model will yield incorrect effect size estimates, and since the confidence intervals around effect sizes are wider in a random effects model, using the wrong model might lead to different conclusions about whether an effect is statistically different from zero or not. For example, Oyserman and Lee [44] use a fixed effect model when performing a meta-analysis of a wide range of individualism vs. collectivism primes, performed by many different labs, using many different dependent variables, and with a significant amount of between-studies variance in the effect sizes. A random effects model should have been used, and the heterogeneity in effect sizes should be explored. If the meta-analyses had been easily reproducible, researchers could have calculated the correct meta-analytic effect size estimate using a random effects model, which then could be used in power analysis when planning new studies.

Identifying erroneously or inconsistently calculated effect sizes requires that reviewers and readers can easily identify the source of the effect size calculation. To improve the low percentage of reproducible findings in meta-analyses (e.g., [30]), a reference to a specific study should preferably be accompanied by a direct quote from the original article describing the test the effect size is derived from (containing information that underlies the coding of study characteristics upon which studies are organized into subgroups, see [55]). If we want to improve the reproducibility of meta-analyses and facilitate quality control, improved disclosure of meta-analytic data is necessary.

Research has shown that the amount of errors meta-analysts make is reduced when the effect sizes are coded by two researchers independently (with disagreements being resolved by a third researcher) than when one person extracts the effect sizes [9]. Authors of meta-analyses need to specify how many people extracted the effect sizes and how disagreements were resolved. Even though such an approach takes more time, it also significantly improves the quality of the meta-analysis.

## Adhere to reporting guidelines

The reporting quality of meta-analyses varies considerably and is often poor [51]. Meta-analyses often insufficiently describe inclusion criteria, the search strategy, or details of how data was extracted from individual papers. This lack of information makes it difficult to assess the strengths and weakness of the meta-analysis. One of the first steps to improve the reporting quality of meta-analyses is using reporting guidelines as a checklist. There are several reporting guidelines available to improve the reporting standards of meta-analyses. One of the most frequently used checklists is the PRISMA statement (Preferred Reporting Items for Systematic Reviews and Meta-Analyses). The PRISMA statement was developed for the health sciences and consists of a two-page checklist and a flow diagram [41]. Within the social sciences there are the (much less widely used) Meta-Analysis Reporting Standards (MARS) from the sixth edition of the APA manual (Publication manual of the [1]) and the MEC2IR reporting standards from the Campbell Collaboration [13]. More reporting guidelines can be found on the website of the EQUATOR network [22]. We recommend that researchers who perform a meta-analysis adhere to proposed reporting standards (such as those specified in the PRISMA statement), while understanding these reporting guidelines will not guarantee reproducibility by themselves.

In recent years, scientists have become more aware of the potential role conflicts of interest can have on the reliability and reproducibility of results. Conflicts of interest have been especially overlooked in meta-analyses, but may have a substantial influence on how meta-analyses are performed. For this reason, some authors have proposed that performing a meta-analysis that includes a substantial number of one’s own studies represents a conflict of interest (for a discussion, see [19]). For example, in a meta-analysis of 90 experiments on precognition [3], studies included in the meta-analysis that were performed by the first author of the meta-analysis all have effect sizes that are equal to or higher than the meta-analytic effect size. Schimmack [53] has noted it is incredibly unlikely any researcher would observe such a set of studies without having additional studies in a file-drawer, which raises the possibility of bias in the inclusion of effect sizes, which at the very minimum should be explicitly addressed.

It is important that authors of meta-analyses take conflicts of interest in the studies they summarize into account when they perform a meta-analysis. It is even more important that authors personally disclose conflicts of interests, which can range from receiving funds from companies who benefit from positive outcomes of a meta-analysis to the fact that authors of a meta-analysis have co-authored a substantial number of the articles included in the meta-analysis. The PRISMA checklist requires researchers to report various possible sources of bias, and invites authors to explain how these conflicts of interest are dealt with, or how they might limit the conclusions that can be drawn from the meta-analysis.

## (Pre-registered) research protocols and prospective meta-analyses

Even though reporting guidelines can improve the quality of reporting after a meta-analysis has been performed, an even better approach is to write a research protocol before the meta-analysis is performed. The goal of such a protocol is to specify how the meta-analysis will be carried out by detailing the search strategy and study selection criteria. Preparing a research protocol can help to uncover some of the pitfalls that could threaten the quality of the meta-analysis later in the process. Typical components of a research protocol include: a specification of the primary and secondary research question(s); a short description of background information about the research topic; details of the search strategy, study selection, and study quality criteria; methods of data extraction; the statistical or qualitative methods that will combine the data; and the project’s timetable ([4]).

Additional advances can be made by making the literature search more transparent and reproducible in both the protocol and in the final article. The reproducibility of the literature search is not only important for quality control, but also to facilitate future updates of the meta-analysis. The literature search is often a major part of the meta-analysis methodology, and just as in other types of research, a well-designed methodology is a requirement for valid results. The current protocols fail to guarantee reproducible literature searches. An attempt by Maggio et al. [40] to reproduce the literature searches of 34 reviews in medical journals revealed not a single literature search could be reproduced. Instead of just reporting which databases were used and which search restrictions were applied, the protocol would benefit from a detailed overview of the full search strategy (either in the main text, or added to the supplementary materials). This includes specifying key concepts with the verbatim search terms that were used to search for these concepts, including the fields that were searched (e.g. title, abstract, subject headings). It should also be clear how the search terms were combined with each other, and which other strategies were employed next to systematic searching (e.g. citation tracking, hand searching, and expert consultation).

Researchers can take research protocols one step further and *pre-register* a research protocol before doing the actual meta-analysis, thereby specifying the planned analysis before the meta-analytic data is extracted. One of the advantages of pre-registration is that it can prevent *confirmation bias* (e.g., deciding which studies to include or exclude based on whether results were in the direction expected or desired by the researcher). Pre-registration allows researchers to clearly differentiate between theory-driven (i.e., based on prior expectations) and data-driven (i.e., based on the outcome of statistical analyses) choices in the meta-analysis.

When a pre-registered meta-analysis is submitted to a journal that reviews submissions before the data is collected (e.g., the “registered report” article format, see [15, 42]), reviewers can comment on the proposed protocol before the data is extracted, instead of submitting commentaries to published meta-analyses after the study has been performed. The use of pre-registered meta-analyses in combination with *in principle acceptance* of pre-registered protocols might reduce the amount of critique raised by other researchers after a meta-analysis is published. Some researchers have doubted whether meta-analyses can actually resolve ideological debates when the selection, coding, and interpretation of studies leaves considerable room for flexibility and interpretation (e.g., [23]). We believe pre-registered protocols for meta-analyses have the potential to considerably improve this situation. Pre-registered meta-analyses in the health sciences can be submitted to PROSPERO [60]. In psychological science, pre-registrations can be performed on websites such as the Open Science Framework. Note that despite common misinterpretations of what pre-registration entails, researchers are free to report and interpret exploratory analyses, as long as it is acknowledged that the Type 1 error rate of these analyses cannot be quantified.

In addition to the (pre-registered) research protocols for meta-analyses, teams of researchers can collaborate to perform a *prospective meta-analysis*. The first prospective meta-analyses in the social sciences are the Many Labs project [38] and the Reproducibility Project [43] in which large teams of researchers collaboratively perform replication studies following a pre-registered protocol. Before the individual results are known, the analysis plan is aimed at meta-analyzing all these datasets. An increasing number of journals in psychology now offer such a pre-registration format. For example, *Perspectives on Psychological Science* recently published its first Registered Replication Report [52], which is also a prospective meta-analyses of a large number of studies using identical methods. As these large-scale replication formats become more common, and with the advent of journals dedicated to pre-registered studies, we can expect prospective meta-analyses to become more common, not only for replication studies, but also for novel theoretical predictions. For example, the new journal *Comprehensive Results in Social Psychology* would allow researchers to test novel theoretical predictions (such as those inspired by opposing theories in adversarial collaborations) and perform a meta-analysis on the results to more quickly and decisively address research questions. Strong predictions from opposing theories can be addressed in a prospective meta-analysis where several labs collaborate, thereby allowing the research team to draw conclusions over a much larger sample size than would be possible in individual studies. Large sample sizes provide high power, even when a relatively strict alpha level is used, and therefore increase the informational value of the statistical inferences [39].

## Facilitating reproducibility

An analysis is reproducible when independent individuals are able to perform the same analysis on the same data. Sharing meta-analytic data does not just facilitate future meta-analyses but also makes it possible for researchers to easily reproduce the published analyses. In addition to directly reproducing the identical analysis, reproducible meta-analyses allow researchers to analyze a subset of the studies included in the meta-analysis, for example, to examine the impact of different inclusion criteria. Researchers could use a subset of the studies to calculate an *a priori* power analysis using the meta-analytic effect size estimate based on a specific subset of studies most similar to the planned research.

Even when inclusion criteria are pre-registered and reviewed by peers, they are necessarily subjective and debatable, not only by researchers with different ideas, but also as new ideas emerge. One example of this subjectivity is the practice of assigning studies to sub-categories which may moderate the strength of the effect examined in meta-analysis. Anderson et al. [2]) coded studies of violent games according to whether they met criteria of “best-practices” in study methodology, which required that participants in the control condition played a game that lacked considerable violence. Application of this criterion includes an element of subjectivity, as definitions of what constitutes a “violent game” vary (see [24]). For example, Anderson and colleagues [2] excluded studies which compared *Mortal Kombat* to *Sonic the Hedgehog* [18, 32] and *Tekken 2* to the racing game *Moto Racer* [8] from the ‘best-practices’ subcategory, while a study in which participants played *Grand Theft Auto 3* or *Simpsons Hit & Run* [6] is included as ‘best practices’ even though the player-character in *Simpsons Hit & Run* drives aggressively and can punch other game characters (earning the game a T-for-Teen ESRB rating). One might disagree with the decisions of the research team, arguing that *Sonic the Hedgehog* lacks violence while *Simpsons Hit & Run* contains violence. If meta-analyses were easily reproducible, researchers could easily explore the impact of these subjective decisions by re-analyzing the meta-analytic data.

Subjective decisions are not limited to the development and application of inclusion criteria. Indeed, meta-analysts often must make subjective decisions in analytic practice among several equally defensible options. In this case, it would be ideal to know how sensitive the results are to these subjective decisions. For example, a recent meta-analysis by Ferguson [25] inspects the partial correlation between violent media and aggressive behavior when other variables are used as covariates. However, the exact partial correlation was not reported by many studies, particularly when the effect did not reach statistical significance. The author decided to impute an effect size *r* = .00 for each unreported, non-significant effect, a justifiable but highly conservative approach (see [5]). Given the controversial nature of this research area, such meta-analyses might be more convincing to ideological opponents when the impact of this imputation can be explored. If this imputation strategy has a large effect on the obtained effect size, the reader can be made aware that the conclusions are highly sensitive to the subjective decisions, and that caution is necessary in interpreting results. On the other hand, if the results are not sensitive to the imputation strategy, the reader can be assured that the results are not contingent on this subjective decision.

Sharing the data underlying the meta-analysis allows readers to evaluate the appropriateness of the inclusion criteria, the creation of sub-categories, or other subjective analytic choices, and see how applying their own criteria might change the results. For example, Jauhar et al. [35] made a spreadsheet document available with their meta-analysis containing all of the analyzed effect sizes. Readers can easily delete (or even add) studies to examine alternative inclusion criteria or sub-categorizations of the same studies. Recent innovations in statistical software (e.g., [27]) make it possible to share all the data underlying a meta-analysis together with the written report (for an excellent example, see [16]). Ideally, the data and analysis are made available using free software such as R (R [47]), which also allows researchers to share the meta-analytical data and analyses (for example, see [11]). At the very minimum, the data is available in a spreadsheet or text file that can be imported in statistical software without considerable effort. It is not sufficient to share data stored in a format that can only be read by a single commercial software package. The Open Science Framework (www.osf.io) and GitHub (www.github.com) are two of many recent technical solutions to facilitate data sharing.

## Recruit expertise

Searching the literature and reporting a reproducible literature search is a skill that requires dedicated knowledge. As the findings by Maggio et al. [40]) reveal, published meta-analyses typically do not include a reproducible search protocol. The research base for performing a systematic search is becoming more and more sophisticated, and we recommend researchers collaborate with expert librarians who are up-to-date with these developments [58]. Formulating a good search strategy is a dynamic and iterative process, which requires knowledge about accessible databases and the syntax required by each database [31]. Researchers often have implicit assumptions about the literature which librarians can either challenge or corroborate [21]. Among other things, this will help to reduce the possibility of bias and improve the overall quality of the search strategy [58]. Furthermore, librarians are experienced at documenting a search strategy for reproducibility purposes, and can advise on which reporting guidelines and quality assessment tools to use, help with writing the methodology section, demonstrate how to pre-register the meta-analysis, and propose services that enable the long-term data storage of all meta-analytic data [46].

Similarly, it is recommended that researchers consult a statistician before coding effect sizes and performing the meta-analysis to make sure their calculations are correct and reproducible. A large percentage of meta-analyses contain errors ([26, 30, 36]). In addition to ensuring that basic effect size extraction and meta-analysis are performed correctly, statisticians can inform researchers about superior meta-analytical techniques such as novel approaches to correct for publication bias. Researchers are often slow to adopt superior statistical techniques when they are applicable (e.g., PET-PEESE meta-regression, see [56]), and continue to use outdated statistical techniques (such as fail-safe *N*, see [50]). It has been suggested that researchers resist novel statistical techniques because they wish to adhere to norms, are unaware of new techniques or cannot perform the new techniques in their preferred statistical software packages [54]. Statisticians, by contrast, might feel less pressure to conform to existing norms in analysis, can introduce researchers to new and superior techniques, and can implement these techniques.

## Conclusion

Meta-analyses are often regarded as state-of-the-art knowledge about effects examined in the scientific literature. At the same time, the conclusions from meta-analyses are often open for debate and are subject to change as new data becomes available. We propose practical recommendations to increase the reproducibility of meta-analyses to facilitate quality control, improve reporting guidelines, allow researchers to re-analyze meta-analyses based on alternative inclusion criteria, and future-proof meta-analyses by making sure the collected meta-analytic data is shared so that continuously cumulating meta-analyses can be performed [7] and so that novel statistical techniques can be applied on the collected data as they become available. Implementing these recommendations will allow us to make great strides towards a truly cumulative science.

Some may object to publically sharing the data underlying a meta-analysis, as they would prefer to have continued proprietary rights to this data after publication in order to publish an updated meta-analysis after several years. We do not believe such ideas are to the benefit of science, nor do they allow appropriate standards of peer-review. Given the large number of errors that are typically observed in meta-analyses ([26, 30, 36]) journals should require authors to publically share the data underlying their analyses. We find that truly cumulative updates of meta-analyses are rare in psychology, a situations that might be improved if the data were publically available. Journals could consider inviting updates of meta-analyses every few years, allowing interested researchers to communicate their interest to collaborate on such an update.

When disclosing all the data underlying a meta-analysis, there are two sources of information that require special care. First of all, the meta-analyst might ask researchers of published studies for additional data or analyses, such as correlations between dependent averages in repeated measure designs, to be able to include a dataset in the meta-analysis. It is important to ask these researchers for permission to disclose this additional information with the published meta-analysis. Second of all, a meta-analysis may include unpublished datasets, and for these an attempt should be made to at least summarize the most important information that underlies the effect size calculation sub-group categorization. If the original authors do not plan to publish the data in the future, the meta-analyst can ask permission to share the unpublished report with the meta-analysis. At the very least, contact information should be provided so that interested parties might obtain a copy of the unpublished report.

Our recommendation to pre-register the research protocol for a meta-analysis should not be misinterpreted as prohibiting researchers to make any changes to the protocol in the course of the research project. One cannot know everything in advance, and writing a research protocol that takes into account all possible future events is extremely difficult. However, it is not necessary to know everything in advance. The goal is to try to consider as much as possible from the outset of the research project. A protocol can and often will need to be updated as the research project progresses, which is perfectly acceptable so long as changes are made before the meta-analytic data are analyzed. Many pre-registered reports can benefit from a more interactive approach to reviews where authors, reviewers and editors communicate changes to the protocol as new insights develop (an approach already applied in Cochrane and Campbell reviews). The main goal of pre-registration is to distinguish theory-driven choices in the analysis plan from data-driven choices that might be biased by the outcome of the meta-analysis, not to prohibit data-driven choices altogether.

Researchers can choose to pre-register their meta-analysis in publically accessible databases, such as PROSPERO, or pre-register (either publically or privately) on the Open Science Framework (www.osf.io) and share the pre-registration with reviewers (and make it publically accessible only after an article has been accepted for publication). One goal of publically accessible pre-registrations is to avoid wasted effort when multiple teams perform meta-analyses of the same research area (although there are benefits in independently reproducing a meta-analysis, as we have argued earlier). Whenever researchers make the effort to reproduce or re-analyze a meta-analysis, they should similarly consider to (publically) pre-register the research protocol, and pursue an in principle acceptance as a registered report. This will prevent only meta-analyses that fail to demonstrate significant effects, or only re-analyses that yield different results than the original meta-analysis, from being published (a publication bias for negative results or successfully reproduced meta-analyses). Obviously, results that confirm the conclusions from the original meta-analysis are equally valuable for cumulative science.

Meta-analyses may be among the most debated scholarly products due to the subjectivity in determining and applying study inclusion criteria, the challenges and ambiguities of extracting effect size estimates from heterogeneous research paradigms, and the profound impact meta-analyses have upon research practice and theory. Transparency of the extracted meta-analytic data is vital for addressing readers’ concerns about the possible presence of errors and the degree to which the conclusions depend on subjective decisions. Disclosure of the meta-analytic data allows researchers to perform other justifiable analyses, including those inspired by new advances in statistical practice. As researchers, data is our livelihood and the currency by which we judge our theories. A truly cumulative science requires reproducible meta-analyses.

## Declarations

### Ethics

Non Applicable.

### Consent to publish

Non Applicable.

### Availability of data and materials

Non Applicable.

## References

[1] American Psychological Association (2009). Publication manual of the American psychological association.

[2] Anderson CA, Shibuya A, Ihori N, Swing EL, Bushman BJ, Sakamoto A, Rothstein HR, Saleem M (2010). Violent video game effects on aggression, empathy, and prosocial behavior in Eastern and Western countries: A meta-analytic review. Psychol Bull.

[3] Bem D, Tressoldi PE, Rabeyron T, Duggan M. Feeling the future: A meta-analysis of 90 experiments on the anomalous anticipation of random future events. 2014. Available at SSRN: http://ssrn.com/abstract=2423692 Accessed 11 Apr 2014. 10.12688/f1000research.7177.1 PMC4706048 26834996

[4] Booth A, Papaioannou D, Sutton A (2011). Systematic approaches to a successful literature review.

[5] Borenstein M, Hedges LV, Higgins JP, Rothstein HR. Introduction to meta-analysis. Chichester, UK: Wiley; 2009.

[6] Brady SS, Matthews KA (2006). Effects of media violence on health-related outcomes among young men. Arch Pediatr Adolesc Med.

[7] Braver SL, Thoemmes FJ, Rosenthal R (2014). Continuously cumulating meta-analysis and replicability. Perspect Psychol Sci.

[8] Brooks MC (2000). Press start: Exploring the effects of violent video games on boys. Diss Abstr Int.

[9] Buscemi N, Hartling L, Vandermeer B, Tjosvold L, Klassen TP (2006). Single data extraction generated more errors than double data extraction in systematic reviews. J Clin Epidemiol.

[10] Bushman BJ, Rothstein HR, Anderson CA (2010). Much ado about something: Violent video game effects and a school of red herring: Reply to Ferguson and Kilburn (2010). Psychol Bull.

[11] Carter EC, McCullough ME. Publication bias and the limited strength model of self-control: has the evidence for ego depletion been overestimated?. Front Psychol. 2014;5. doi:10.3389/fpsyg.2014.00823. 10.3389/fpsyg.2014.00823 PMC4115664 25126083

[12] The Campbell Collaboration. Campbell Systematic Reviews: Policies and Guidelines. Campbell Syst Rev 2014. 2014a;Supplement 1. http://dx.doi.org/10.4073/csrs.2014.1.

[13] The Campbell collaboration. Methodological Expectations of Campbell Collaboration Intervention Reviews (MEC2IR) DRAFT Version 0.1, June 23, 2014 (Updated September 2014). 2014. Retrieved from: http://www.campbellcollaboration.org/Methods_Resources/MEC2IR.php.

[14] Campbell L, Loving TJ, LeBel EP, Enhancing Transparency of the Research Process to Increase Accuracy of Findings: A Guide for Relationship Researchers. 2014. Available at SSRN: http://ssrn.com/abstract=2435185 or http://dx.doi.org/10.2139/ssrn.2435185.

[15] Chambers CD, Feredoes E, Muthukumaraswamy SD, Etchells P (2014). Instead of “playing the game” it is time to change the rules: Registered Reports at AIMS Neuroscience and beyond. AIMS Neurosci.

[16] Churches T. The benefits of reproducible research: a public health example. 2013. Retrieved from https://github.com/timchurches/meta-analyses/blob/master/benefits-of-reproducible-research/benefits-of-reproducible-research.md.

[17] The Cochrane Collaboration. Cochrane Handbook for Systematic Reviews of Interventions (Version 5.1.0), Higgins J, Green S, editors. 2011. Retrieved from http://www.cochrane-handbook.org. Accessed 11 Apr 2014.

[18] Cohn LB (1995). Violent video games: Aggression, arousal, and desensitization in young adolescent boys.

[19] Coyne JC, Kok RN (2014). Salvaging psychotherapy research: a manifesto. J Evid-Based Psychotherapies.

[20] Derry CJ, Derry S, McQuay HJ, Moore RA (2006). Systematic review of systematic reviews of acupuncture published 1996–2005. Clin Med.

[21] Dudden RF, Protzko SL (2011). The systematic review team: contributions of the health sciences librarian. Med Ref Serv Q.

[22] EQUATOR Network. Library for health research reporting. 2014. Retrieved from: http://www.equator-network.org/library/. Accessed, 21 Oct 2014.

[23] Ferguson CJ (2014). Comment: Why meta-analyses rarely resolve ideological debates. Emot Rev.

[24] Ferguson CJ. Violent video games don’t exist. The Huffington Post. 2014. Retrieved from http://www.huffingtonpost.com/christopher-j-ferguson/violent-video-games-dont-_b_5051733.html. Accessed 11 Apr 2014.

[25] Ferguson CJ. Do Angry Birds make for angry children? A meta-analysis of video game influences on children’s and adolescents’ aggression, mental health, prosocial behavior and academic performance. Perspect Psychol Sci. (in press). 10.1177/1745691615592234 26386002

[26] Ford AC, Guyatt GH, Talley NJ, Moayyedi P (2009). Errors in the conduct of systematic reviews of pharmacological interventions for irritable bowel syndrome. Am J Gastroenterol.

[27] Gandrud C. Reproducible research with R and RStudio. CRC Press. 2013.

[28] Goodyear-Smith FA, van Driel ML, Arroll B, Del Mar C (2012). Analysis of decisions made in meta-analyses of depression screening and the risk of confirmation bias: a case study. BMC Med Res Methodol.

[29] Gorin SS, Krebs P, Badr H, Janke EA, Jim HS, Spring B, Jacobsen PB. Meta-analysis of psychosocial interventions to reduce pain in patients with cancer. J Clin Oncol. 2012;30(5);539-47. JCO-2011. 10.1200/JCO.2011.37.0437 PMC6815997 22253460

[30] Gøtzsche PC, Hróbjartsson A, Marić K, Tendal B (2007). Data extraction errors in meta-analyses that use standardized mean differences. JAMA.

[31] Harris MR (2005). The librarian’s roles in the systematic review process: a case study. J Med Libr Assoc.

[32] Hoffman, K. Effects of playing versus witnessing video game violence on attitudestoward aggression and acceptance of violence as a means of conflict resolution.Dissertation Abstracts International. 1995;56(03):747. (University Microfilms No. 9522426)

[33] Horton J, Vandermeer B, Hartling L, Tjosvold L, Klassen TP, Buscemi N (2010). Systematic review data extraction: cross-sectional study showed that experience did not increase accuracy. J Clin Epidemiol.

[34] Iyengar S, Greenhouse JB (1988). Selection models and the file drawer problem. Stat Sci.

[35] Jauhar S, McKenna PJ, Radua J, Fung E, Salvador R, Laws KR (2014). Cognitive-behavioural therapy for the symptoms of schizophrenia: systematic review and meta-analysis with examination of potential bias. Br J Psychiatry.

[36] Jones AP, Remmington T, Williamson PR, Ashby D, Smyth RL (2005). High prevalence but low impact of data extraction and reporting errors were found in Cochrane systematic reviews. J Clin Epidemiol.

[37] Journal Article Reporting Standards Working Group (2008). Reporting standards for research in psychology: Why do we need them? What might they be?. Am Psychol.

[38] Klein RA, Ratliff KA, Vianello M, Adams RB, Bahnik S, Bernstein MJ, Nosek BA (2014). Investigating variation in replicability: A “Many Labs” replication project. Soc Psychol.

[39] Lakens D, Evers ER (2014). Sailing From the Seas of Chaos Into the Corridor of Stability Practical Recommendations to Increase the Informational Value of Studies. Perspect Psychol Sci.

[40] Maggio LA, Tannery NH, Kanter SL (2011). Reproducibility of literature search reporting in medical education reviews. Acad Med.

[41] Moher D, Liberati A, Tetzlaff J, Altman DG, The PRISMA Group (2009). Preferred Reporting Items for Systematic Reviews and Meta-Analyses: The PRISMA Statement. PLoS Med.

[42] Nosek BA, Lakens D (2014). Registered Reports. Soc Psychol.

[43] Open Science Collaboration (2012). An open, large-scale, collaborative effort to estimate the reproducibility of psychological science. Perspect Psychol Sci.

[44] Oyserman D, Lee SW (2008). Does culture influence what and how we think? Effects of priming individualism and collectivism. Psychol Bull.

[45] Peters JL, Sutton AJ, Jones DR, Abrams KR, Rushton L (2007). Performance of the trim and fill method in the presence of publication bias and between‐study heterogeneity. Stat Med.

[46] Pinfield S, Cox AM, Smith J (2014). Research data management and libraries: relationships, activities, drivers and influences. PLoS One.

[47] R Core Team. R: A language and environment for statistical computing. R Foundation for Statistical Computing, Vienna, Austria. ISBN 3-900051-07-0. 2012. URL http://www.R-project.org/. Accessed 11 Apr 2014

[48] Rothstein HR, Sutton AJ, Borenstein M, editors. Publication bias in meta-analysis: Prevention, assessment and adjustments. Wiley; 2006.

[49] Rouder JN, Morey RD (2011). A Bayes factor meta-analysis of Bem’s ESP claim. Psychon Bull Rev.

[50] Scargle JD (2000). Publication bias: the “file-drawer” problem in scientific inference. J Scientific Explor.

[51] Sacks HS, Reitman D, Pagano D, Kupelnick B (1995). Meta-analysis: an update. The Mount Sinai J Med, New York.

[52] Simons DJ, Holcombe AO, Spellman BA (2014). An Introduction to Registered Replication Reports at Perspectives on Psychological Science. Perspect Psychol Sci.

[53] Schimmack U (2012). The ironic effect of significant results on the credibility of multiple-study articles. Psychol Methods.

[54] Sharpe D (2013). Why the resistance to statistical innovations? Bridging the communication gap. Psychol Methods.

[55] Simonsohn U, Nelson LD, Simmons JP (2014). *P*-curve: A key to the file-drawer. J Exp Psychol Gen.

[56] Stanley TD, Doucouliagos H (2014). Meta-regression approximations to reduce publication selection bias. Res Synth Methods.

[57] Sutton AJ, Abrams KR (2001). Bayesian methods in meta-analysis and evidence synthesis. Stat Methods Med Res.

[58] Swinkels A, Briddon J, Hall J (2006). Two physiotherapists, one librarian and a systematic literature review: collaboration in action. Health Inf Libr J.

[59] Terrin N, Schmid CH, Lau J, Olkin I (2003). Adjusting for publication bias in the presence of heterogeneity. Stat Med.

[60] University of York, Centre for Reviews and Dissemination. PROSPERO. International prospective register of systematic reviews. 2014. http://www.crd.york.ac.uk/PROSPERO/. Accessed 11 Apr 2014.

[61] De Wit R, van Dam F, Zandbelt L, van Buuren A, van der Heijden K, Leenhouts G, Loonstra S (1997). A pain education program for chronic cancer pain patients: follow-up results from a randomized controlled trial. Pain.

[62] De Wit R, Van Dam F (2001). From hospital to home care: a randomized controlled trial of a Pain Education Programme for cancer patients with chronic pain. J Adv Nurs.

[63] De Wit R, van Dam F, Loonstra S, Zandbelt L, Buuren A, Heijden K, Abu‐Saad HH (2001). Improving the quality of pain treatment by a tailored pain education programme for cancer patients in chronic pain. Eur J Pain.

